# A review of the main genetic factors influencing the course of COVID-19 in Sardinia: the role of human leukocyte antigen-G

**DOI:** 10.3389/fimmu.2023.1138559

**Published:** 2023-06-05

**Authors:** Stefano Mocci, Roberto Littera, Luchino Chessa, Marcello Campagna, Maurizio Melis, Carla Maria Ottelio, Ignazio S. Piras, Sara Lai, Davide Firinu, Stefania Tranquilli, Alessia Mascia, Monica Vacca, Daniele Schirru, Luigi Isaia Lecca, Stefania Rassu, Federica Cannas, Celeste Sanna, Mauro Giovanni Carta, Francesca Sedda, Erika Giuressi, Selene Cipri, Michela Miglianti, Andrea Perra, Sabrina Giglio

**Affiliations:** ^1^ Medical Genetics, Department of Medical Sciences and Public Health, University of Cagliari, Cagliari, Italy; ^2^ AART-ODV (Association for the Advancement of Research on Transplantation), Cagliari, Italy; ^3^ Medical Genetics, R. Binaghi Hospital, Local Public Health and Social Care Unit (ASSL) of Cagliari, Cagliari, Italy; ^4^ Department of Medical Sciences and Public Health, University of Cagliari, Cagliari, Italy; ^5^ Liver Unit, University Hospital, Cagliari, Italy; ^6^ Anesthesia and Intensive Care Unit, R. Binaghi Hospital, Local Public Health and Social Care Unit (ASSL) of Cagliari, Cagliari, Italy; ^7^ Neurogenomics Division, Translational Genomics Research Institute (TGen), Phoenix, AZ, United States; ^8^ Section of Pathology, Oncology and Molecular Pathology Unit, Department of Biomedical Sciences, University of Cagliari, Cagliari, Italy; ^9^ GeneMos-APS (Association for Social Advancement), Reggio Calabria, Italy; ^10^ Centre for Research University Services (CeSAR, Centro Servizi di Ateneo per la Ricerca), University of Cagliari, Monserrato, Italy

**Keywords:** COVID-19, Sardinian population, soluble HLA-G, HLA-G 3’UTR haplotypes, KIR2DS2 gene, neanderthal LZTFL1 variants

## Abstract

**Introduction:**

A large number of risk and protective factors have been identified during the SARS-CoV-2 pandemic which may influence the outcome of COVID-19. Among these, recent studies have explored the role of HLA-G molecules and their immunomodulatory effects in COVID-19, but there are very few reports exploring the genetic basis of these manifestations. The present study aims to investigate how host genetic factors, including *HLA-G* gene polymorphisms and sHLA-G, can affect SARS-CoV-2 infection.

**Materials and Methods:**

We compared the immune-genetic and phenotypic characteristics between COVID-19 patients (n = 381) with varying degrees of severity of the disease and 420 healthy controls from Sardinia (Italy).

**Results:**

HLA-G locus analysis showed that the extended haplotype HLA-G*01:01:01:01/UTR-1 was more prevalent in both COVID-19 patients and controls. In particular, this extended haplotype was more common among patients with mild symptoms than those with severe symptoms [22.7% *vs* 15.7%, OR = 0.634 (95% CI 0.440 – 0.913); P = 0.016]. Furthermore, the most significant *HLA-G 3’UTR* polymorphism (*rs371194629*) shows that the *HLA-G 3’UTR Del/Del* genotype frequency decreases gradually from 27.6% in paucisymptomatic patients to 15.9% in patients with severe symptoms (X^2 ^= 7.095, P = 0.029), reaching the lowest frequency (7.0%) in ICU patients (X^2 ^= 11.257, P = 0.004). However, no significant differences were observed for the soluble HLA-G levels in patients and controls. Finally, we showed that SARS-CoV-2 infection in the Sardinian population is also influenced by other genetic factors such as β-thalassemia trait (*rs11549407*C>T in the *HBB* gene), *KIR2DS2/HLA*-C C1+ group combination and the HLA-B*58:01, C*07:01, DRB1*03:01 haplotype which exert a protective effect [P = 0.005, P = 0.001 and P = 0.026 respectively]. Conversely, the Neanderthal *LZTFL1* gene variant (*rs35044562*A>G) shows a detrimental consequence on the disease course [P = 0.001]. However, by using a logistic regression model, *HLA-G 3’UTR Del/Del* genotype was independent from the other significant variables [OR_M_ = 0.4 (95% CI 0.2 – 0.7), P_M_ = 6.5 x 10^-4^].

**Conclusion:**

Our results reveal novel genetic variants which could potentially serve as biomarkers for disease prognosis and treatment, highlighting the importance of considering genetic factors in the management of COVID-19 patients.

## 1 Introduction

Over the past 20 years, human coronaviruses (HCoVs), such as SARS-CoV, MERS-CoV, and SARS-CoV-2, have resulted in outbreaks of serious respiratory illness (1–6). The severe acute respiratory syndrome coronavirus 2 (SARS-CoV-2) has caused the ongoing coronavirus disease-19 (COVID-19) pandemic (7–10), which has led to more than 6.3 million deaths globally as of June 2022 (11–13). COVID-19 can result in severe respiratory distress syndrome, coagulopathies, septic shock, and multiple organ injuries (14–17). Studies have revealed differences in COVID-19 incidence and lethality based on gender and age, with a higher incidence in women and higher lethality in men (18). Additionally, young individuals (aged 0-24) have a lower COVID-19 incidence rate than a group of people over 65 years of age (19, 20). Vaccine development has appeared to be the most effective approach in slowing the spread of COVID-19 (21–26), but the emergence of new variants of concern (VOC) has challenged vaccine efficacy and durability (27–30).

Various clinical outcomes have been described among COVID-19 patients since the outbreak. While some patients remain asymptomatic, others may develop respiratory or multiorgan failure with potentially lethal outcomes (31, 32).

Genetic factors may influence the individual’s susceptibility or resistance to viral infections by regulating the immune response (33–38). In particular, recent studies have also highlighted the role of specific genetic variants associated with asymptomatic COVID-19, such as genes of the lectin pathway (39).

However, the majority of the studies have indicated that COVID-19 development and/or severity are associated with polymorphisms in innate and adaptive immune genes, including killer-cell immunoglobulin-like receptors (*KIR*) and human leukocyte antigens (*HLA*) class I and II as well as genes involved in viral response pathways (*LZTFL1, OAS* gene family) (40–43).

The HLA-G, a non-classical HLA class I molecule, has been shown to play a critical role in immune response modulation and has been implicated in various pathological processes including response to viral infections (44). It is physiologically expressed in extravillous cytotrophoblast cells and is an essential factor in maternal-fetal tolerance (45, 46). Given its central role in immunotolerance, it is involved in several pathological conditions such as carcinogenesis, acute and chronic inflammation, autoimmune diseases, organ transplantation, allergies, parasitic diseases and response to viral infections (47, 48). HLA-G molecules interact primarily with ILT-2/LILRB1, ILT-4/LILRB2 and KIR2DL4 receptors, the immune checkpoint, to exert its inhibitory effects on immune cells (45). The interaction of HLA-G molecules with these specific receptors inhibits the proliferation and maturation of dendritic cells, cytotoxic NK cells (CD56dim, CD16+), and induces apoptosis in CD8+ T cells, while reducing the proliferation of CD4+ T cells and B cells (49). Thus, HLA-G can interfere in many different immunological processes of both the innate and adaptive immune system leading to a reduced immune response. Because of the extensive number of alleles and their associated regulatory regions (IPD-IMGT/HLA database, version 3.24.0.1), HLA-G expression levels between individuals differ widely (50). Moreover, the complexity of this system is increased by regulation at the transcriptional and post-translational levels, resulting in the production of seven alternative transcripts, four of which are membrane-bound (HLA-G1-G4), and 3 are soluble (HLA-G5-G7) (51, 52). To date, there were found several single nucleotide polymorphisms (SNPs) and an *HLA-G* insertion/deletion (*Ins/Del; rs3711944629*) of 14-base pair on the 3’UTR, in which the *Del-Del* genotype has been associated with high expression of HLA-G mRNA, whereas the *Ins-Ins* genotype has been associated with lower mRNA production (45, 49, 53). Several haplotypes have been described in the 3’UTR (*UTR-1, UTR-2, UTR-3, UTR-4, UTR-5, UTR-6/-18, and UTR-7)* of this gene, suggesting that HLA-G may influence immune responses to different stimuli, including in viral infections (45). Although the exact mechanisms by which the immunomodulatory molecule HLA-G influences disease presentation and progression are still not fully understood, research has shown that viral infections can lead to an increase in both the cell surface membrane-bound and soluble peripheral expression of HLA-G (53).

The association between HLA-G expression and COVID-19 severity and progression has been studied in some studies with contradictory results. Two hypotheses have been proposed to explain this discrepancy: the first is that the immunosuppressive action of HLA-G enhances the virus’s ability to escape the immune system, while the second is that HLA-G expression and secretion are a robust response to inflammation during the viral infection (53, 54). This suggests that high levels of HLA-G molecules may inhibit neutrophil adhesion to endothelial cells, resulting in a negative association between elevated levels of HLA-G and disease progression (55, 56).

In most of the studies conducted to date, the attention has focused mainly on the expression of HLA-G and serum levels of the molecule in patients with severe COVID-19, rather than the genetic basis from which these manifestations result. Starting from this consideration, this study aims to investigate the genetic basis of the *HLA-G* and its role in the manifestation of SARS-CoV-2 infections. To this aim, we used the Sardinian population as a model of study, which is noteworthy for its high degree of genetic homogeneity. This makes it ideal for studying genetic and immunogenetic features, including the role of innate and adaptive immunity in viral infections.

In previous studies, carried out during the outbreak of the SARS-CoV-2- B.1.1.7 variant in Italy, we observed how specific genetic factors in the Sardinian population significantly impact the outcome of COVID-19 infections (36, 38, 43). We have found that certain genetic factors, including the *HLA* extended haplotype HLA-B58:01, C07:01, DRB1*03:01, β°39 C>T variant at the *HBB* gene and *KIR2DS2* gene/HLA C1 group ligand combination, can positively influence the course of the disease and result in an asymptomatic outcome (36, 38, 43). While the Neanderthal-inherited haplotype (*rs35044562*, *rs73064425*, *rs34326463*, *rs67959919*) at the leucine zipper transcription factor-like 1 (*LZTFL1*) has been associated with an increased risk of serious symptoms (36).

In this study, we evaluated a new and larger group of unvaccinated individuals affected by COVID-19, who were enrolled during the spread of the B.1.617.1 (Delta) variant (13). This allowed us to investigate whether the previously identified risk and protective factors still played a role in the infection caused by this different SARS-CoV-2 variant. Moreover, we explored other genetic traits, such as *OAS3* protective haplotype and G6PDH enzyme deficiencies, which have not been found critical in our past studies (36, 38).

This study aimed to confirm the robustness of previous findings with a new pool of individuals and investigate the role of *HLA-G* in COVID-19. In particular, we evaluated the role of *HLA-G*, both as a single factor and in correlation with other factors, to determine its strength in affecting the outcome of the disease. Throughout this study, the goal was to contribute to the development of a broader spectrum of prognostic factors that could be used in the future.

## 2 Materials and methods

### 2.1 Patients and controls selection

In this study, we analyzed data from 381 unvaccinated patients, recruited between 1 August and 30 October 2021 at the Covid Unit of the SS.Trinità Hospital in Cagliari (Italy). In this period, the predominant variant circulating worldwide was the SARS-CoV-2 Delta (B.1.617.2) (25). All the recruited patients were diagnosed with SARS-CoV-2 by RT-PCR from a nasopharyngeal swab. Following the WHO’s guidelines, patients were divided into two groups: 207 patients had been admitted to the Covid Unit of the SS.Trinità Hospital in Cagliari with moderate or severe disease, including 57 intensive care unit (ICU) patients, (Group S) and 174 asymptomatic or paucisymptomatic patients (Group A) were confined to home quarantine. Four hundred twenty unrelated healthy individuals, from the Sardinian Voluntary Bone Marrow Donor Registry, were enrolled as control group. According to three-generation family trees, both groups (patients and controls) were from South Sardinia.

### 2.2 Ethics statement

The research protocol was conducted at the Department of Medical Sciences and Public Health of the University of Cagliari, the University Hospital of Cagliari (AOUCA), and the SS. Trinità Hospital of the Sardinian Regional Company for the Protection of Health (ATS Sardegna) where patient recruitment took place. In accordance with the local human research committee’s national and institutional ethical standards, all patients and controls provided informed consent. The informed consent procedures in the study protocol are in accordance with the ethical guidelines outlined in the Declaration of Helsinki and have been approved by the responsible ethics committee (Ethics Committee of the Cagliari University Hospital; date of approval: May 27th, 2020; protocol number GT/2020/10894). Documents containing written informed consent are kept on file and included in each patient’s clinical records.

### 2.3 DNA extraction and genetic analysis

The genomic DNA from peripheral blood mononuclear cells was extracted following the standard methods (57). All 801 samples from patients and controls were genotyped at high resolution for the alleles at *HLA-A, -B, -C, -DR* and -*G* loci using Next-generation sequencing (NGS) AlloSeq Tx17 (CareDx) method based on Hybrid Capture Technology and performed on the Illumina platform. The data was analyzed using the AlloSeq Assign^®^ software (v.1.0.2). The full-length *HLA-G* gene was sequenced through long-range PCR, including the 3’UTR non-coding region. Primers were designed using Primer3web (version 4.1.0), based on HLA-G RefSeqGene version NG_029039.1 (NCBI database), as previously described (58).

Starting with 1 ng of PCR product, the libraries were prepared using the Nextera XT DNA Library Preparation Kit. On MiSeq Illumina Sequencer, a pool of normalized libraries (4 nM) was loaded onto V3 flow cells for 600 cycles of paired-end sequencing. Alignment and variant calling of the FASTQ files were processed by MiSeq Reporter v2.6, and variant classification was performed using VariantStudio Software v3.0 (Illumina, Netherlands). Each variant was validated individually and then entered into appropriate spreadsheets for statistical analysis as reported later on.

The 3’UTR haplotypes of *HLA-G* were determined based on variations in their nucleotide sequences between +2945 and +3259 nucleotides of the 3’UTR using the methodology and nomenclature described elsewhere (47, 59–61).

Moreover, we performed the *KIR* typing in order to detect the presence of the 14 *KIR* genes *KIR2DL1, KIR2DL2, KIR2DL3, KIR2DL4, KIR2DL5, KIR3DL1, KIR3DL2, KIR3DL3, KIR2DS1, KIR2DS2, KIR2DS3, KIR2DS4, KIR2DS5* and *KIR3DS1* using PCR-SSP with primers specific for each locus according to a previously reported method (62, 63).

We explored the Neanderthal haplotype in the *LZTFL1* gene, which consists of the variants *rs35044562, rs73064425, rs34326463, rs67959919*, and is most strongly associated with the risk of developing a severe form of COVID-19. We considered *rs35044562* as the index risk variant for severe infection (36).

Additionally, we examined another Neanderthal inherited variant located in the *OAS3* gene (*rs1156361*) which has been associated with protection against severe COVID-19 (64). Our final step was to sequence the *rs11549407* (C>T) variant at codon 39 of the hemoglobin subunit beta gene (*HBB*), the predominant mutation responsible for beta-thalassemia in Sardinia (65). Primer pairs for each region of interest were designed using Primer3web (version 4.1.0), as we previously reported (36).

The PCR reaction was performed according to the protocol supplied with AmpliTaq Gold™ DNA Polymerase (Applied Biosystems/Thermo Fisher Scientific, Waltham, MA) for each region containing the three SNPs: *rs35044562*, *rs1156361* and *rs11549407* respectively located within the *LZTFL1, OAS3* and the *HBB* gene.

Sequencing was performed using the BigDye™ Terminator v3.1 Cycle Sequencing Kit (Applied Biosystems, USA), with the same primers described previously followed by cleanup with CleanSEQ Dye-Terminator Removal Kit (Beckman Coulter, Inc.). Capillary electrophoresis was run on the ABI 3500 Genetic Analyzer (Applied Biosystems) and sequences were analyzed with Sequencher 5.3 (^©^ 2017 Gene Codes Corporation).

### 2.4 Soluble HLA-G and G6PD activity quantification

Plasma samples were collected from all 381 convalescent COVID-19 patients, from one to six months after recovery, and 420 controls were recruited for this study. The levels of sHLA-G were determined using the sHLA-G ELISA assay kit (Exbio, Prague, Czech Republic), which detects both shedding HLA-G-1 and soluble HLA-G-5 molecules. The assay was conducted on the plasma samples immediately frozen after separation and stored at -80°C until use. Fifty µl of each sample were diluted 1:80 in the plasma-specific buffer. A six-point calibration curve was obtained using the human native HLA-G protein included in the kit. At the end of the reaction, optical density was measured using a microplate reader with a 450 nm filter. The limit of sensitivity was 0.6 U/ml. For all samples a technical replicate was included.

The activity of the G6PD enzyme was quantified by measuring an increase in absorbance of NADPH at 340 nm during the enzyme-catalyzed reaction (66). The assay was conducted using the Randox G6PD assay kit (Randox Laboratories Ltd., Crumlin, UK) as described in other studies (67).

### 2.5 Statistical analysis

Clinical and biochemical parameters of COVID-19 patients were reported using mean values and standard deviations (SD) or percentages, as appropriate. The Student’s t-test was used to compare continuous variables between patients and controls. The Fisher’s exact test was used to determine P values and odds ratios (ORs), along with their 95% confidence intervals (CIs), when we compared the categorical data between patients and controls or subgroups of patients. To account for multiple testing based on the number of *HLA-G* alleles or 3’UTR haplotypes analyzed, the P values (Pc) were adjusted. The results were considered statistically significant only when the adjusted P value (Pc) was lower than 0.05. Statistical analysis was performed with the R programming language (R version 4.2.2) [R core Team (2022). R: A language and environment for statistical computing. R Foundation for Statistical Computing, Vienna, Austria. URL https://www.R-project.org/]. We examined the Hardy-Weinberg equilibrium (HWE) of the *HLA-G* 14bp *Ins/Del* polymorphism by computing ${\text{X}}_{\text{HWE}}^{2}$ and P values. Deviation from HWE was assessed using Haploview 4.0 software (Broad Institute, Cambridge, MA, USA) (68).

All tests were two-sided and only values of P < 0.05 were accepted as being statistically significant.

Soluble HLA-G plasma levels in controls, patients and subgroups of patients were represented by boxplots. We used the non-parametric Wilcoxon rank sum test (69) for comparisons between two groups and the Kruskal-Wallis rank sum test (70) for comparisons between three groups.

A multivariate logistic regression analysis was conducted to determine the independence of clinical and genetic variables with respect to age and gender, based on the results of the univariate analysis (P_U_< 0.05) that showed statistically significant differences between the groups (A and S patients). The univariate P values and ORs have adjusted accordingly to age and gender using a logistic regression model. The multivariate P values (P_M_) were corrected for multiple comparisons (P_MC_).

## 3 Results

### 3.1 Clinical characteristics of patients with COVID-19

Clinical and genetic characteristics of SARS-CoV-2 positive patients are shown in **Table 1**. The mean age at diagnosis was 56 years (mean ± SD: 56.0 ± 17.4). According to the results, 28.4% (n = 108) of the patients were under the age of 50, and 41.5% were over 65 years of age.

**Table 1:** Comparisons of baseline clinical and genetic parameters between COVID-19 patients with either asymptomatic/pauci-symptomatic (Group A) or moderate/severe (Group S) disease.

Characteristics of Sardinian COVID-19 patients	Total pts N = 381		Group A N = 174		Group S N = 207		Comparison Group S *vs* Group A	
Age (yr): mean ± SD	56.0 ± 17.40		51.97 ± 17.47		62.43 ± 16.99		*P* = 1.3·10^-11^	
	n	%	n	%	n	%	*P* value	OR (95% CI)
Age ≤ 50 yr	108	0.2835	82	0.4713	26	0.1256	**5.3·10^-14^**	0.2 (0.1 – 0.3)
50 yr< Age< 65 yr	115	0.3018	47	0.2701	68	0.3285	0.221	1.3 (0.8 – 2.1)
Age ≥ 65 yr	158	0.4147	45	0.2586	113	0.5459	**1.1·10^-7^**	3.4 (2.2 – 5.3)
Male	226	0.5932	72	0.4138	154	0.7440	**6.2·10^-11^**	4.1 (2.7 – 6.4)
Female	155	0.4068	102	0.5862	53	0.2560	**6.2·10^-11^**	0.2 (0.2 – 0.4)
Comorbidities								
Cancer	6	0.0157	4	0.0230	2	0.0097	0.418	0.4 (0.1 – 2.3)
Diabetes	13	0.0341	4	0.0230	9	0.0435	0.397	1.9 (0.6 – 6.4)
Chronic pulmonary disease^1^	5	0.0131	3	0.0172	2	0.0097	0.663	0.6 (0.1 – 3.4)
Ischemic heart disease^2^	28	0.0735	15	0.0862	13	0.0628	0.433	0.7 (0.3 – 1.5)
Hypertension	57	0.1496	25	0.1437	32	0.1546	0.776	1.1 (0.6 – 1.9)
Autoimmune disease^3^	75	0.1969	24	0.1379	51	0.2464	**0.009**	2.0 (1.2 – 3.5)
Hypercholesterolemia	38	0.0997	14	0.0805	24	0.1159	0.304	1.5 (0.8 – 3.0)
Chronic Medication use								
Steroidal anti-inflammatory drug	20	0.0525	5	0.0287	15	0.0725	0.066	2.6 (0.9 – 7.4)
Non steroidal anti-inflammatory drug^4^	22	0.0577	7	0.0402	15	0.0725	0.194	1.9 (0.7 – 4.7)
ACE II inhibitor^5^	39	0.1024	15	0.0862	24	0.1159	0.398	1.4 (0.7 – 2.7)
Angiotensin II receptor blocker^6^	21	0.0551	9	0.0517	12	0.0580	0.826	1.1 (0.5 – 2.7)
Beta and calcium channel blockers^7^	48	0.1260	20	0.1149	28	0.1353	0.643	1.2 (0.7 – 2.2)
Levothyroxine	23	0.0604	14	0.0805	9	0.0435	0.138	0.5 (0.2 – 1.2)
Genetic trait								
Beta-thalassemia Trait^8^	22	0.0577	18	0.1034	4	0.0193	**0.001**	0.2 (0.1 – 0.5)
G6PDH deficiency	40	0.1050	16	0.0920	24	0.1159	0.504	1.3 (0.7 – 2.5)
*LZTFL1* (rs35044562)^9^	76	0.1995	22	0.1264	54	0.2609	**0.001**	2.4 (1.4 – 4.2)
*OAS3* (*rs1156361*)^10^	339	0.6378	152	0.6207	187	0.6522	0.412	1.3 (0.7 – 2.6)
HLA-B*58:01, C*07:01, DRB1*03:01 ^11^	8	0.0210	7	0.0402	1	0.0048	**0.026**	0.1 (0.0 – 0.9)
*KIR AA* Haplotype	127	0.3333	55	0.3161	72	0.3478	0.586	1.2 (0.7 – 1.8)
*KIR2DS2*/HLAC C1+ group ligand	110	0.2887	63	0.3621	47	0.2271	**0.005**	0.5 (0.3 – 0.8)

^1^ Chronic obstructive pulmonary disease was defined as a diagnosis of emphysema and/or bronchitis.

^2^ Ischemic heart disease was categorized as history of myocardial infarction or angina

^3^Autoimmune diseases included Hashimoto’s thyroiditis, type I diabetes mellitus, rheumatoid arthritis, systemic lupus erythematosus and autoimmune hepatitis

^4^ Non-steroidal anti-inflammatory drugs included aspirin, ibuprofen, diclofenac, naproxen, indomethacin, celecoxib, and meloxicam.

^5^ Angiotensin converting enzyme inhibitors included captopril, enalapril, lisinopril, fosinopril, ramipril, and quinapril.

^6^ Angiotensin II receptor blockers included losartan, candesartan, irbesartan, olmesartan, and valsartan.

^7^ Dihydropyridine calcium channel blockers included amlodipine, nifedipine. Beta blockers included atenolol, bisoprolol, labetalol, metoprolol, nebivolol.

^8^ The variant at codon 39 (C>T, rs11549407) in the HBB gene has been found in more than 90% of beta thalassemia carrier in Sardinia and has been associated with protection against severe COVID-19.

^9^ The allelic variant rs35044562 (A>G) in the LZTFL1 gene, inherited from Neanderthal. has been associated with the highest risk of severe infection of SARS-CoV-2 (36).

^10^ The allelic variant rs1156361 (T>C) in the OAS3 gene, inherited from Neanderthal, has been associated with protection against severe COVID-19 (70).

^11^ None of the patients with SARS-CoV-2 infection carried the four loci extended haplotype HLA-A*02:05, B*58:01, C*07:01, DRB1*03:01 which was present in 3.1% of the 420 people selected for the Sardinian population group control [OR = 0.1 (95% CI 0.0 – 0.6), P = 0.002].

P values were calculated for comparisons between Sardinian COVID-19 patients, Group A vs Group S. SD, standard deviation; CI, confidence interval. The bold values mean statistical significance. Bold formatting are used to highlight values that have achieved statistical significance based on the applied tests or criteria.

A significant proportion of patients over 65 years of age had more severe symptoms and clinical manifestations than adults less than 65 years old [OR = 3.4 (95% CI 2.2–5.3), P = 1.1 x 10^−7^].

On the other hand, a significant percentage of patients under the age of 50 seemed to have less severe symptoms than patients over 50 years old [OR = 0.2 (95% CI 0.1–0.3), P = 5.3 x 10^−8^].

COVID-19 occurred at the same rate in males and females, with males slightly more likely to contract the disease (59.3%). In addition, the results suggest that female patients are less susceptible to COVID-19 severe effects [74.4% males *vs*. 25.6% females, OR = 4.1 (95% CI 2.7–6.4), P = 6.2 x 10^-11^].

Our analysis also included several other comorbidities that may be involved in the disease course of COVID-19, in addition to sex and age. In our cohort, most patients with comorbidity had autoimmune diseases (19.7%). The remaining comorbidities were: 15% arterial hypertension, 10% hypercholesterolemia, 7.36% ischemic heart disease, 3.5% type I diabetes, 1.3% chronic pulmonary disease and 1.1% cancer. For each comorbidity present in the total cohort of patients, we compared group S (severe) and group A (asymptomatic) patients. There was no significant difference regarding comorbidities except for a higher prevalence of autoimmune disorders among patients with severe disease [24.6% A *vs*. 13.8% S, OR = 2.0 (95% CI 1.2–3.5), P = 0.009]. Moreover, there was no difference in chronic drug intake between the two groups of patients.

### 3.2 Genetic traits influencing COVID-19 outcome

As a further step, we investigated genetic traits likely to influence the outcome of COVID-19 caused by the B.1.617.2 SARS-CoV-2 variant in the Sardinian population. The results of each genetic trait analyzed are presented in detail in **Supplementary Tables** (S1: Allele and Genotype distribution of *rs35044562*, *rs1156361* and *rs11549407* in SARS-CoV-2 patients; S2: *HLA* alleles and Haplotypes frequencies compared between Group A and S; S3: *KIR* genes and genotype frequencies compared between Group A and S; S4: Comparisons of *KIR* genes and their cognate HLA ligands between COVID-19 patients between Group A and S).

None of the patients with SARS-CoV-2 infection in group S carried the extended haplotype HLA-A*02:05, B*58:01, C*07:01, DRB1*03:01 whose frequency was 3.2% in the control group [OR 0.1 (95% CI 0.1 – 0.6), P = 0.002].

The three-locus *HLA* haplotype HLA-B*58:01, C*07:01, DRB1*03:01 was almost absent in group S [2.0% A *vs* 0.48% S, OR = 0.1 (95% CI 0.0 – 0.9), P = 0.026], as well as the *KIR2DS2/*HLA-C C1+ group ligand, which was more prevalent in group A [36.2% A *vs* 22.7% S, OR = 0.5 (95% CI 0.3 – 0.8), P = 0.005], suggesting their protective effect.

Similar results were obtained for the beta-thalassemia trait (*rs11549407* C>T) that was found in 5.7% of patients, with higher prevalence in group A than in group S [10.3% A *vs*. 1.9% S, OR = 0.2 (95% CI 0.1 – 0.5), P = 0.001].

Additionally, the Neanderthal variant *rs35044562* (A>G), reported to be associated with a severe form of COVID-19, was clearly associated with group S [12.6% A *vs*. 26.1% S, OR = 2.4 (95% CI 1.4 - 4.2), P = 0.001]. Finally, there was no significant difference between the two groups of patients in terms of frequencies in the *KIR AA* haplotype, G6PDH enzyme deficiency and *OAS3* (*rs1156361*) polymorphism [62% A *vs* 65% S, OR = 1.1 (95% CI 0.8 – 1.7), P = 0.593].

#### 3.2.1 Analysis of the locus HLA-G

##### 3.2.1.1 A comparison of HLA-G alleles and 3’UTR haplotype frequencies between the population group and COVID-19 patients

*HLA-G* alleles and 3’UTR haplotype frequencies were compared in 381 COVID-19 patients and 420 healthy controls (**Table 2**). The analysis of extended haplotypes (*HLA-G* alleles and 3’UTR haplotype) showed there were few substantive differences in frequency between patients and healthy controls. In both groups, the most prevalent extended haplotypes were HLA-G*01:01:01:01/*UTR-1* (27.5% controls, 18.9% patients), HLA-G*01:03:01:02/UTR-5 (15.6% controls, 9.8% patients), and HLA-G*01:01:02:01/UTR-2 (11.8% controls, 13.8% patients).

**Table 2:** Extended haplotypes (HLA-G alleles and 3’UTR haplotypes) frequencies in healthy controls and COVID-19 patients.

HLA-G extended haplotypes		N = 420 Healthy controls		N = 381 COVID-19 patients		Controls *vs* Patients		
Alleles	3’UTR haplotype	2N = 840	%	2N = 762	%	P value^§^	OR (95% CI)	*Pc*
G*01:01:01:01	UTR-1	231	0.2750	144	0.1890	< 0.001	1.6 (1.3 – 2.1)	**0.002**
G*01:03:01:02	UTR-5	131	0.1560	75	0.0984	0.001	1.7 (1.3 – 2.3)	**0.018**
G*01:01:02:01	UTR-2	99	0.1179	105	0.1378	0.260	0.8 (0.6 – 1.1)	
G*01:01:03:03	UTR-7	57	0.0679	72	0.0945	0.054	0.7 (0.5 – 1.0)	
G*01:01:01:08	UTR-1	55	0.0655	63	0.0827	0.213	0.8 (0.5 – 1.1)	
G*01:01:22:01	UTR-2	54	0.0643	51	0.0669	0.840	1.0 (0.6 – 1.4)	
G*01:01:01:03/05	UTR-4	42	0.0500	59	0.0774	0.030	0.6 (0.4 – 0.9)	
G*01:04:01:01	UTR-3	46	0.0548	87	0.1142	< 0.001	0.5 (0.3 – 0.7)	**0.005**
G*01:06:01:01/02	UTR-2	36	0.0429	39	0.0512	0.478	0.8 (0.5 – 1.3)	
G*01:05N	UTR-2	15	0.0179	27	0.0354	0.029	0.5 (0.3 – 0.9)	
G*01:04:04	UTR-3	16	0.0190	9	0.0118	0.314	1.6 (0.6 – 3.7)	
G*01:01:01:04	UTR-18	15	0.0179	3	0.0039	0.008	4.6 (1.3 – 16.0)	
G*01:04:01:01	UTR-10	9	0.0107	0	0.0000	0.004	–	
G*01:01:01:06	UTR-4	6	0.0071	1	0.0013	0.127	5.5 (0.7 – 45.6)	
G*01:04:01:02	UTR-3	6	0.0071	0	0.0000	0.032	–	
G*01:01:01:01	UTR-4	3	0.0036	0	0.0000	0.251	–	
G*01:01:01:04	UTR-6	3	0.0036	18	0.0236	0.001	0.1 (0 – 0.5)	**0.018**
G*01:01:01:03	UTR-7	0	0.0000	3	0.0039	0.107	0.0 (0.0 – 2.2)	
G*01:01:02:01	UTR-13	0	0.0000	3	0.0039	0.107	0.0 (0.0 – 2.2)	
G*01:01:01:06	UTR-2	3	0.0036	0	0.0000	0.251	–	
G*01:06:02:02	UTR-2	3	0.0036	1	0.0013	0.626	2.7 (0.3 – 26.3)	
G*01:01:01:01	UTR-3	1	0.0012	0	0.0000	1.000	–	
G*01:03:01:01	UTR-5	4	0.0048	0	0.0000	0.126	–	
G*01:02:02	UTR-2	3	0.0036	0	0.0000	0.251	–	
G*01:01:01:09	UTR-1	2	0.0024	0	0.0000	0.501	–	
G*01:01:17	UTR-2	0	0.0000	2	0.0026	0.226	0.0 (0.0 – 4.8)	

^§^ P values were calculated for comparisons between Sardinian COVID-19 patients and the population group. Pc corresponds to P values corrected for multiple comparisons. OR, odds ratio; CI, confidence interval; % = allele frequencies expressed as decimals. The bold values mean statistical significance. Bold formatting are used to highlight values that have achieved statistical significance based on the applied tests or criteria.

The HLA-G*01:01:01:01/UTR-1 and HLA-G*01:03:01:02/UTR-5 showed a lower and significantly different frequency in patients [(27.5% *vs* 18.9%, OR = 1.6 (95% CI 1.3 – 2.1); P< 0.001, P_c_ = 0.002) and [15.6% *vs* 9.8%, OR = 1.7 (95% CI 1.3 – 2.3); P = 0.001, P_c_ = 0.018)].

Conversely, the HLA-G*01:04:01:01/UTR-3 and HLA-G*01:01:01:04/*UTR-6* were more prevalent in the patient’s group (5.5% *vs* 11.4% and 0.36% *vs* 2.4% respectively), with a significant difference only for the first of the two haplotypes: HLA-G*01:04:01:01/*UTR-3* [OR = 0.5 (95% CI 0.3 – 0.7); P< 0.001, Pc = 0.005].

Additionally, we evaluated the frequencies of *HLA-G* 3’UTR haplotypes (**Table 3**). In both groups, UTR-1 was the most prevalent haplotype in both groups. Additionally, only *UTR-1* and *UTR-5* showed a significant difference in frequency between controls and patients [34.3% *vs* 27.2%, OR = 1.4 (95% CI 1.1 – 1.7); P = 0.002, Pc = 0.02 and 16.1% *vs* 9.8%, OR = 1.8 (95% CI 1.3 – 2.4); P< 0.001, P_c_ = 0.001 respectively]. *UTR-6* and *UTR-10* showed marginal significance in their P-values due to their low frequencies.

**Table 3:** Haplotype frequencies observed at the HLA-G 3’UTR polymorphic sites in population group controls and COVID-19 patients.

HLA-G 3’UTR	N = 420 controls		N = 381 Covid-19 patients		Controls *vs* Patients		
Haplotypes	2N = 840	%	2N = 762	%	^§^ *P*-value	OR (95% CI)	Pc
UTR-1 (DelTGCCCGC)	288	0.3429	207	0.2717	0.002	1.4 (1.1 – 1.7)	**0.02**
UTR-2 (InsTCCCGAG)	213	0.2536	225	0.2953	0.064	0.8 (0.7 – 1.0)	
UTR-5 (InsTCCTGAC)	135	0.1607	75	0.0984	< 0.001	1.8 (1.3 – 2.4)	**0.001**
UTR-3 (DelTCCCGAC)	69	0.0821	96	0.1260	0.005	0.6 (0.4 – 0.9)	
UTR-7 (InsTCATGAC)	57	0.0679	75	0.0984	0.029	0.7 (0.5 – 1.0)	
UTR-4 (DelCGCCCAC)	51	0.0607	60	0.0787	0.168	0.8 (0.5 – 1.1)	
UTR-18 (DelTGCCCAC)	15	0.0179	3	0.0039	0.008	4.6 (1.3 – 16.0)	
UTR-10 (DelTCCCGAG)	9	0.0107	0	0.0000	0.004	–	0.042
UTR-6 (DelTGCCCAC)	3	0.0036	18	0.0236	0.001	0.1 (0.1 – 0.5)	**0.017**
UTR-13 (DelTCCTGAC)	0	0.0000	3	0.0039	0.107	0.0 (0.0 – 2.2)	
UTR-8 (InsTGCCGAG)	0	0.0000	0	0.0000	–	–	

Analysis of eight polymorphisms at the 3′UTR of HLA-G [hg38 chr6:29,827,845-29,831,125]. Haplotype combination of polymorphisms at the 3′UTR of HLA-G: HLA-G 14bp Ins/Del (rs3711944629), 3003C>T (rs1707), 3010C>G (rs116152775), 3027A>C (rs17179101), 3035C>T (rs17179108), 3142C>G (rs1063320), 3187A>G (rs9380142), and 3196C>G (rs1610696). ^§^P values were calculated for comparisons between Sardinian COVID-19 patients and the population group. Pc corresponds to P values corrected for multiple comparisons. OR, odds ratio; CI, confidence interval; % = allele frequencies expressed as decimals. The bold values mean statistical significance. Bold formatting are used to highlight values that have achieved statistical significance based on the applied tests or criteria.

##### 3.2.1.2 Correlation of HLA-G alleles and 3’UTR haplotype frequencies to the clinical manifestations of SARS-CoV-2 infection

The patients were then divided into two groups based on disease severity: Group A (asymptomatic and paucisymptomatic ill SARS-CoV-2 patients) and Group S (severely ill patients).

The results of the analysis of *HLA-G* alleles and *3’UTR* haplotype frequencies in patients with SARS-CoV-2 infection appeared notable in relation to the severity of the clinical picture (**Table 4**). Among the twenty-six *HLA-G* extended haplotypes, only the HLA-G*01:01:01:01/*UTR-1* and HLA-G*01:01:03:03/UTR-7 showed a significant association. In particular, the extended haplotype HLA-G*01:01:01:01/UTR-1 was more frequent in Group A [22.7% *vs* 15.7%, OR = 0.634 (95% CI 0.440 – 0.913); Pc = 0.016] (**Table 5**).

**Table 4:** Comparisons of HLA-G extended haplotypes among COVID-19 patients divided according to severity of the clinical manifestations.

HLA-G extended haplotypes		N = 381 COVID-19 patients		N = 174 Group A (paucisymptomatic)		N = 207 Group S (severe symptoms)		Group A *vs* Group S	
Alleles	3’UTR haplotype	2N = 762	%	2N = 348	%	2N = 414	%	P value^§^	OR (95% CI)
G*01:01:01:01	UTR-1	144	0.189	79	0.227	65	0.157	0.016	0.634 (0.440 – 0.913)
G*01:03:01:02	UTR-5	75	0.098	27	0.078	48	0.116	0.087	1.559 (0.951 – 2.557)
G*01:01:02:01	UTR-2	105	0.138	56	0.161	49	0.118	0.093	0.700 (0.463 – 1.058)
G*01:01:03:03	UTR-7	72	0.094	21	0.060	51	0.123	0.004	2.188 (1.288 – 3.716)
G*01:01:01:08	UTR-1	63	0.083	32	0.092	31	0.075	0.429	0.799 (0.477 – 1.339)
G*01:01:22:01	UTR-2	51	0.067	21	0.060	30	0.072	0.505	0.822 (0.462 – 1.464)
G*01:01:01:03/05	UTR-4	59	0.077	27	0.078	32	0.077	0.988	1.004 (0.589 – 1.711)
G*01:04:01:01	UTR-3	87	0.114	36	0.103	51	0.123	0.393	0.821 (0.522 – 1.291)
G*01:06:01:01/02	UTR-2	39	0.051	15	0.043	24	0.058	0.354	0.732 (0.378 – 1.418)
G*01:05N	UTR-2	27	0.035	15	0.043	12	0.029	0.294	1.509 (0.697 – 3.268)
G*01:04:04	UTR-3	9	0.012	3	0.009	6	0.014	0.455	0.591 (0.147 – 2.382)
G*01:01:01:04	UTR-18	3	0.004	1	0.003	2	0.005	0.667	0.594 (0.054 – 6.575)
G*01:04:01:01	UTR-10	0	0	–	0	–	0	0.931	1.190 (0.024 – 60.11)
G*01:01:01:06	UTR-4	1	0.001	1	0.003	0	0	0.605	2.386 (0.080 – 71.34)
G*01:04:01:02	UTR-3	0	0	–	0	–	0	–	
G*01:01:01:01	UTR-4	0	0	–	0	–	0	–	
G*01:01:01:04	UTR-6	18	0.024	8	0.023	10	0.024	0.916	0.951 (0.371 – 2.435)
G*01:01:01:03	UTR-7	3	0.004	2	0.006	1	0.002	0.464	2.387 (0.216 – 26.44)
G*01:01:02:01	UTR-13	3	0.004	1	0.003	2	0.005	0.667	0.594 (0.051 – 6.575)
G*01:01:01:06	UTR-2	0	0	–	0	–	0	0.931	1.190 (0.024 – 60.11)
G*01:06:02:02	UTR-2	1	0.001	1	0.003	0	0	0.605	2.386 (0.080 – 71.34)
G*01:01:01:01	UTR-3	0	0	–	0	–	0	–	
G*01:03:01:01	UTR-5	0	0	–	0	–	0	–	
G*01:02:02	UTR-2	0	0	–	0	–	0	–	
G*01:01:01:09	UTR-1	0	0	–	0	–	0	–	
G*01:01:17	UTR-2	2	0.003	2	0.006	0	0	0.275	4.786 (0.215 – 106.48)

^§^ P values were calculated for comparisons between Sardinian COVID-19 patients, Group A vs Group S. OR, odds ratio; CI, confidence interval. Group A: asymptomatic or paucisymptomatic patients, Group S: patients with severe disease.

**Table 5:** Haplotype frequencies observed at the HLA-G 3’UTR polymorphic sites in COVID-19 patients divided according to severity of the clinical manifestations.

HLA-G 3’UTR	381 COVID-19 patients		174 Group A (paucisymptomatic)		207 Group S (severe symptoms)		Group A *vs* Group S		
Haplotypes	2N = 762	%	2N = 348	%	2N = 414	%	P^§^	OR (95% CI)	*Pc*
UTR-1 (DelTGCCCGC)	207	0.272	112	0.3218	95	0.2295	0.005	0.628 (0.455 – 0.865)	0.045
UTR-2 (InsTCCCGAG)	225	0.295	108	0.3103	117	0.2826	0.403	0.875 (0.641 – 1.120)	
UTR-5 (InsTCCTGAC)	75	0.098	27	0.0776	48	0.1159	0.087	1.559 (0.951 – 2.557)	
UTR-3 (DelTCCCGAC)	96	0.126	39	0.1121	57	0.1377	0.289	0.790 (0.512 – 1.221)	
UTR-7 (InsTCATGAC)	75	0.098	24	0.0690	51	0.1232	0.014	1.897 (1.142 – 3.151)	0.126
UTR-4 (DelCGCCCAC)	60	0.079	27	0.0776	33	0.0797	0.914	0.971 (0.572 – 1.650)	
UTR-18 (DelTGCCCAC)	3	0.004	1	0.0029	2	0.0048	1.000	1.684 (0.152 – 18.656)	
UTR-10 (DelTCCCGAG)	0	0.000	0	0.0000	0	0.0000	–		
UTR-6 (DelTGCCCAC)	18	0.024	9	0.0259	9	0.0217	0.709	1.195 (0.469 – 3.044)	
UTR-13 (DelTCCTGAC)	3	0.004	1	0.0029	2	0.0048	0.667	0.594 (0.054 – 6.575)	
UTR-8 (InsTGCCGAG)	0	0.00	0	0.0000	0	0.0000	–		

Analysis of eight polymorphisms at the 3′UTR of HLA-G [hg38 chr6:29,827,845-29,831,125]. Haplotype combination of polymorphisms at the 3′UTR of HLA-G: HLA-G 14bp Ins/Del (rs3711944629), 3003C>T (rs1707), 3010C>G (rs116152775), 3027A>C (rs17179101), 3035C>T (rs17179108), 3142C>G (rs1063320), 3187A>G (rs9380142), and 3196C>G (rs1610696). ^§^P values were calculated for comparisons between Sardinian COVID-19 patients and the population group. Pc corresponds to P values corrected for multiple comparisons. OR, odds ratio; CI, confidence interval. Group A: asymptomatic or paucisymptomatic patients, Group S: patients with severe disease. % = allele frequencies expressed as decimals.

In addition, we examined the genotype and alleles frequencies distribution of the *HLA-G 14bp Ins/Del* polymorphism in both controls and patients (**Table 6**). Among the nine polymorphisms constituting the *HLA-G UTR-1* haplotype, the *Del* variant (*rs371194629*, 14bp deletion) was found to be the most significant. The *Ins/Del* polymorphism variants revealed a distribution in Hardy–Weinberg equilibrium (HWE) both in group A patients and the control population. The ${\text{X}}_{\text{HWE}}^{2}$ P-values for the control population were not statistically significant ( ${\text{X}}_{\text{HWE}}^{2}$ = 0.789, P = 0.375 and ${\text{X}}_{\text{HWE}}^{2}$ = 1.031, P = 0.310, respectively). On the other hand, the *HLA-G* 14bp *Ins/Del* polymorphism in the total COVID-19 patient population and in Group S were not in HWE ( ${\text{X}}_{\text{HWE}}^{2}$ = 8.527, P = 0.003 and ${\text{X}}_{\text{HWE}}^{2}$ = 10.369, P = 0.001, respectively). Finally, the HWE for Intensive Care Unit (ICU) patients was close to being statistically significant ( ${\text{X}}_{\text{HWE}}^{2}$ = 3.079, P = 0.079).

**Table 6:** HLA-G 14bp polymorphism in COVID-19 patients and population group control.

	Alleles (f)						Genotypes (f)					
	n	Allele 2n	Ins n (%)	Del n (%)	HWE X^2^	HWE (P value)	Ins/Ins n (%)	Ins/Del n (%)	Del/Del n (%)	X^2^	P value	
Population group control	420	840	411 (0.489)	429 (0.511)	0.789	0.375	96 (0.229)	219 (0.521)	105 (0.250)		
COVID-19 patients	381	762	381 (0.500)	381 (0.500)	**8.527**	0.003*	81 (0.213)	219 (0.574)	81 (0.213)	2.475	0.290
• Group A	174	348	159 (0.457)	189 (0.543)	1.031	0.310	33 (0.190)	93 (0.534)	48 (0.276)	1.217	0.544
• Group S	207	414	222 (0.536)	192 (0.464)	**10.369**	0.001*	48 (0.232)	126 (0.609)	33 (0.159)	**7.095**	**0.029^#^**
− ICU patients	57	114	74 (0.649)	40 (0.351)	3.079	0.114*	21 (0.369)	32 (0.561)	4 (0.070)	**11.257**	**0.004^§^**

Group A = asymptomatic and paucisymptomatic patients, Group S = patients with severe clinical manifestation, ICU patients = patients admitted to Intensive Care Units, HWE = Hardy–Weinberg Equilibrium.

* Allele frequencies of Ins and Del variants were not in HWE in COVID-19 patients Group S. Allele frequencies of ICU were on the edge of significance. ^#^ P value was calculated for comparison between the genotype of HLA-G (Ins/Ins, Ins/Del, Del/Del) in Group S vs Population group control. ^§^ P value was calculated for comparison between the genotype of HLA-G (Ins/Ins, Ins/Del, Del/Del) in ICU patients vs Population group control. The bold values mean statistical significance. Bold formatting are used to highlight values that have achieved statistical significance based on the applied tests or criteria.

The deviation from HWE is due to the reduced frequency of *HLA-G Del* variants among severe COVID-19 patients. The frequency of *HLA-G Del/Del* genotype decreases gradually from 27.6% in Group A to 15.9% in Group S (X^2 ^= 7.095, P = 0.029), and reaches its lowest frequency of 7.0% among ICU patients (X^2 ^= 11.257, P = 0.004).

**Figure 1:**
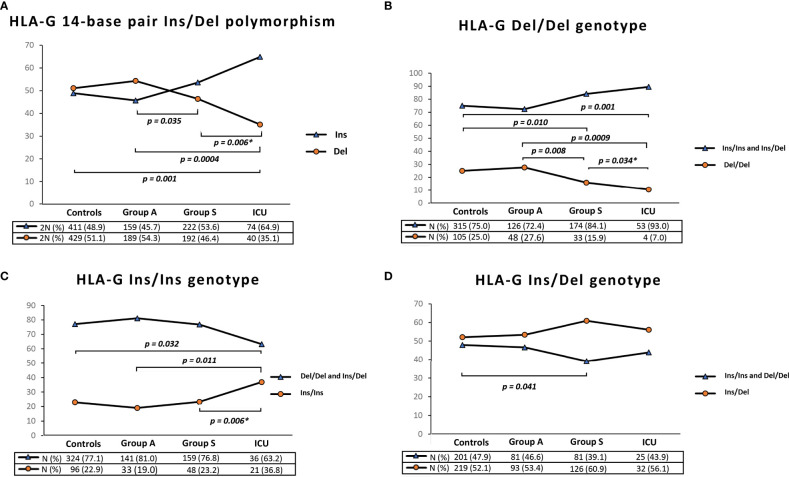
Graphical representation of *HLA-G 3’*UTR 14bp *Ins/Del* allele frequency **(A)**, *HLA-G* 3’UTR 14bp *Del/Del* genotype frequency **(B)**, *HLA-G* 3’UTR 14bp *Ins/Ins* genotype frequency **(C)** and *HLA-G* 3’UTR 14bp *Ins/Del* genotype frequency. Data extracted from controls, Group A (Paucisymptomatic patients), Group S (patients with severe symptoms) and ICU (critical patients admitted in Intensive Care Unit). P values were calculated by using the two-tailed Fisher’s exact test. Only P values less than 0.05 are reported in the figure corresponding to significant differences between the frequencies of the *HLA-G* 3’U*TR* 14-bp polymorphism (*Ins or Del*) and the *HLA-G* genotype (Del/Del or Ins/Ins and Ins/Del) in the control sample and in the groups of patients. **Table S5** in the **Supplementary Material** reports the P values for all the possible comparisons between the HLA-G polymorphism and genotypes in the groups of controls and patients. *To calculate the P values between Group S and ICU, we excluded the patients in ICU from Group S.

The 14bp *Ins/Del* polymorphism at *HLA-G* 3’UTR implies an imbalance between *Ins* and *Del* variants, particularly in severe and ICU patients. As shown in **Figure 1A** *Del* allele was significantly less frequent in Group S and ICU patients compared to Group A and controls. Specifically, the *Del* variant was present in 35.1% (2N=40) of ICU patients and in 51.1% (2N= 429) of controls (ICU *vs* controls, P = 0.001) and in 54.3% (2N=189) of Group A (ICU *vs* Group A, P = 0.0004).

Similarly, we observed a significant difference between Group S and Group A [46.4% (2N=192) *vs* 54.3% (2N=189), P = 0.035] and between Group S and ICU patients [35.1% (2N=40) *vs* 46.4% (2N=192), P = 0.006].

Based on genotype frequencies for the *HLA-G* 14-bp (**Figure 1B**), *Del/Del* genotype was significantly less common in Group S and in ICU patients [N= 33 (15.9%) and N=4 (7%), respectively] compared to Group A and controls [N= 48 (27.6%) and N=105 (25.0%), respectively] (**Figure 1B**).

On the other hand, we found the opposite situation for the *Ins/Ins* genotype. As shown in **Figure 1C**, this *HLA-G 3’UTR* genotype was significantly more frequent in ICU patients [N=21 (36.8%)] compared to Group S [N=48 (23.2%)], Group A [N=33 (19.0%)] and controls [N=96 (22.9%)].

Finally, the frequency of the *Ins/Del* genotype in Group S was slightly higher than in controls [N=126 (60.9%) *vs* N=219 (52.1%), P=0.041] (**Figure 1D**). **Table S5** displays all the P values from the comparison of all groups.

##### 3.2.1.3 Soluble HLA-G dosage

Soluble HLA-G (sHLA-G) levels were measured in healthy controls at the time of enrollment, and in patients at a time point between one to six months after complete recovery. The levels of sHLA-G were not measured in plasma obtained during the acute disease or during hospitalization because sHLA-G levels might be influenced by the pharmacologic treatment that was not standardized.

Results show that sHLA-G levels are similar between COVID-19 recovered patients and controls (**Supplementary Figure** **S1A**), with no statistically significant differences between the two groups [sHLA-G: Median (IQR) 5.9 (7.2) *vs*. 7.5 (8) U/mL P = 0.068]. We obtained the same results when we compared Group A *vs*. Group S (**Supplementary Figure** **S1B**), [sHLA-G: Median (IQR) 5.5 (4.8) *vs*. 7.3 (8.1) U/mL P = 0.960].

According to the literature, serum levels of HLA-G may be affected by the presence of *Del* allele. Patients were therefore divided into the genotype group: homozygous (*Ins/Ins*), heterozygous (*Ins/Del*) and homozygous (*Del/Del*). However, no significant differences were found between the three groups (**Supplementary Figure** **S1C**), [sHLA-G: Median (IQR) 5.3 (4.3) *Ins/Ins vs* 4.6 (7.2) *Ins/Del vs* 6.9 (8.2) *Del/Del* U/mL, P = 0.429].

Finally, we evaluated the effect of the *Del/Ins* genotype in Group A and Group S. No differences were found in this case either [Group A: Median (IQR) 4.6 (4.4) *Ins/Ins vs* 5.6 (5.4) *Ins/Del vs*. 5.7 (3.6) *Del/Del* U/mL P = 0.406] and [Group S: Median (IQR) 5.9 (2.4) *Ins/Ins vs* 4.3 (9.7) *Ins/Del vs*. 11.5 (4.6) *Del/Del* U/mL P = 0.368] (**Supplementary Figures** **S1D, E** respectively).

### 3.3 Multivariate analysis of clinical, immunological and genetic factors and the clinical manifestations of SARS-CoV-2 infection

As a further analysis, we performed a multivariate analysis based on a logistic regression model to calculate the independence of immunological and genetic variables from age and gender.

We included in the comparisons between the two groups of patients (A and S) the factors that were statistically significant in the univariate analysis (P_U_< 0.05): concomitant autoimmune diseases, beta-thalassemia trait, *HLA-G 3’ UTR 14bp Del/Del*, the KIR-ligand combination *KIR2DS2/*HLA-C C1+, the Neanderthal *LZTFL1* polymorphism and the three loci HLA haplotype HLA-B*58:01, C*07:01, DRB1*03:01. The univariate P values and odds ratios of the statistically significant factors were adjusted according to age and gender (**Table 7**). The multivariate P values (P_M_) were corrected for multiple comparisons (P_MC_).

**Table 7:** Multivariate analysis of clinical, immunological and genetic factors associated with the course of the SARS-CoV-2 disease.

Characteristics of Sardinian Covid-19 pts	Total pts (N = 381)		Group A (N = 174)		Group S (N = 207)		Comparison between group S and group A						
							Univariate Analysis			Multivariate analysis			
	n	%	n	%	n	%	OR	95% CI	P_U_^^^	OR_M_^#^	95% CI_M_^§^	P_M_^^^	P_MC_^^^
Age ≥ 65 yr	138	36.2	49	28.2	89	42.3	1.9	1.2 – 3.0	0.003	2.5	1.6 – 4.1	**1.3·10^-4^**	**0.001**
Male gender	226	59.3	72	41.4	154	74.4	4.1	2.6 – 6.5	6.2·10^-11^	4.8	3.1 – 7.7	**1.8·10^-11^**	**1.4·10^-10^**
Autoimmune disease^&^	75	19.7	24	13.8	51	24.6	2.0	1.2 – 3.7	0.009	2.4	1.3 – 4.3	**0.004**	**0.032**
*HBB rs11549407* (C>T)	22	5.8	18	10.3	4	1.9	0.17	0.04 – 0.53	6.0·10^-4^	0.2	0.0 – 0.5	**0.002**	**0.016**
HLA-G 3’ UTR 14bp *Del/Del*	84	22.0	52	29.9	32	15.5	0.4	0.3 – 0.7	8.0·10^-4^	0.4	0.2 – 0.7	**6.5·10^-4^**	**0.005**
*KIR2DS2*/HLA C1 combination +^⁑^	110	28.9	63	36.2	47	22.7	0.5	0.3 – 0.8	0.005	0.6	0.4 – 0.9	**0.031**	0.248
*LZTFL1 rs35044562* (A>G)	76	19.9	22	12.6	54	26.1	2.4	1.4 – 4.4	0.001	2.2	1.2 – 4.0	**0.010**	0.080
HLA-B*58:01, C*07:01, DRB1*03:01 ^†^	8	2.1	7	4.0	1	0.5	0.1	0.0 – 0.9	0.026	0.1	0.0 – 0.8	0.060	0.480

Multivariate analysis based on a logistic regression model included all the clinical, immunological and genetic variables found significantly associated (P value ≤ 0.05 in univariate analysis) with the course of the viral infection: age ≥ 65 yr, male gender, concomitant autoimmune diseases, β-thalassemic trait rs11549407 HBB gene (C>T), Neanderthal LZTFL1 gene rs35044562 (A>G), KIR-ligand combination KIR2DS2/HLA C1 and the HLA-G 3’UTR 14 bp Del/Del genotype. In the comparisons between Group S and Group A, age and gender were the most relevant factors, therefore the odds ratios of all variables were adjusted accordingly. ^^^ P_U_ = P value in univariate analysis; P_M_ = P value in multivariate analysis based on a logistic regression model; P_MC_ = multivariate P value corrected for multiple comparisons by the Bonferroni method.

^#^ OR_M_ = Odds ratio adjusted for age and gender; ^§^ 95% CI_M_ = 95% confidence interval calculated using the logistic regression model.

^&^ Rheumatoid arthritis, type I diabetes mellitus and autoimmune hepatitis; ^⁑^+ = present. ^†^ The result for the three loci HLA haplotype (HLA-B*58:01, C*07:01, DRB1*03:01) is not fully reliable since logistic regression analysis is a large sample method which requires at least 10 subjects. The bold values mean statistical significance. Bold formatting are used to highlight values that have achieved statistical significance based on the applied tests or criteria.

The results confirmed the strong association between the severe clinical manifestations of SARS-CoV-2 infection and five clinical and genetic factors after multiple testing correction, as shown in **Table 7**: I) age ≥ 65 years [OR_M_ = 2.5 (95% CI 1.6 – 4.1), P_M_ = 1.3 x 10^-4^, P_MC_ = 0.001], II) male gender [OR_M_ = 4.8 (95% CI 3.1 – 7.7), P_M_ = 1.8·10^-11^, P_MC_ = 1.4 x 10^-10^], III) autoimmune disease [OR_M_ = 2.4 (95% CI 1.3 – 4.3), P_M_ = 0.004, P_MC_ = 0.032], IV) β-Thalassemia trait [OR_M_ = 0.2 (95% CI 0.0 – 0.5), P_M_ = 0.002, P_MC_ = 0.016] and V) *HLA-G* 3’ UTR 14bp *Del/Del* [OR_M_ = 0.4 (95% CI 0.2 – 0.7), P_M_ = 6.5 x 10^-4^, P_MC_ = 0.005]. The results confirmed that this specific *HLA-G 3’UTR* polymorphism plays a relevant role in protection against severe and life-threatening diseases.

## 4 Discussion

The clinical course of SARS-CoV-2 infection can be influenced by various clinical, genetic, and immunological factors, and may also depend on the specific variant of the virus, as observed in various studies. Since the first wave of the pandemic emerged, males and patients of advanced age have been more susceptible to severe clinical manifestations (71).

In the Sardinian population as well, these two clinical-demographic factors have been identified as the most relevant risk factors (43). Nevertheless, this study highlights additional clinical and genetic factors that may impact the progression of SARS-CoV-2 B.1.617.1 (Delta) variant infection among Sardinian population, beyond those previously identified during the spread of the B.1.1.7 variant (36, 43).

Among these factors, comorbidity with autoimmune diseases emerged as the most relevant clinical factor. The four most common autoimmune diseases associated with severe COVID-19 were Hashimoto’s thyroiditis, type I diabetes, rheumatoid arthritis, systemic lupus erythematosus, and autoimmune hepatitis.

According to the literature, patients with these immune-mediated diseases have an alteration of the cell-mediated immune response mechanism that facilitates a rapid release of proinflammatory cytokines and chemokines by T lymphocytes, resulting in the so-called “cytokine storm” that complicates COVID-19 course (9, 38, 43, 72).

Our study, in addition to these comorbidities, highlights the association of the Neanderthal haplotype at the *LZTFL1* gene with severe COVID-19.

This haplotype, consisting of four SNPs (*rs35044562, rs73064425, rs34326463, rs67959919*) has exerted a negative influence on disease outcome. The findings are in line with previous studies in other populations showing that this Neanderthal haplotype is strongly linked to a severe form of COVID-19 (36, 73, 74).

The biological role of *LZTFL1* in COVID-19 outcomes is still unclear. However, it should be noted that *LZTFL1* gene expression is widely distributed in pulmonary epithelial cells, including those of the ciliated epithelium, which has been identified as a major target for SARS-CoV-2 infections. This gene encodes a cytosolic leucine zipper protein, which associates with the epithelial marker E-cadherin and participates in wound healing and immune response. According to previous studies, increased expression of *LZTFL1* caused by a gain-of-function variant in an inducible enhancer may negatively affect the outcome in COVID-19 patients (75).

In contrast, according to previous studies in different populations, we confirmed that the other Neanderthal haplotype encompassing the *OAS1, OAS2*, and *OAS3* genes (chr12: 113,350,796 to 113,425,679; hg19) does not provide any protection against severe SARS-CoV-2 infections in Sardinia population (65, 76, 77).

Another notable observation that emerged from this study was the low frequency of beta-thalassemia carriers in the group of patients with severe clinical manifestations. In particular, the most common mutation in Sardinia, the β°39 C>T (*rs11549407*), at the gene *HBB*, does not protect against infection but appears to enhance resistance in cases of severe disease.

Currently, the mechanism conferring resistance to severe COVID-19 infection remains to be elucidated. According to some studies, microRNAs produced by patients with hemoglobinopathies are involved in modulating the functions associated with several disease processes, including microbial defense (78).

Other researchers suggested that specific SARS-CoV-2 proteins may attack the heme on the 1-ß chain of hemoglobin, leading to iron dissociation, formation of porphyrin, and consequent oxidative damage (79).

This molecular process could be less frequent in patients with hemoglobinopathies. Nevertheless, this result needs to be further investigated by future studies since the literature does not provide unique data.

On one side, some studies have found the protective effect of the thalassemia trait and hemoglobinopathies (43, 78, 80). Other studies, however, reject this conclusion and suggest an increased mortality risk from COVID-19 in patients with hemoglobinopathies (81). This inconsistency is likely caused by the wide variety of mutations found in alpha and beta globin chains that are characteristic of different populations.

As a result of multivariate analysis, it was also confirmed that the *KIR2DS2/*HLA-C C1+ group-ligand combination can influence the outcome of COVID-19 disease independently of other genetic factors. During SARS-CoV-2 infection, the number of circulating NK cells decreases, and the cells express markers of exhaustion (TIM-3, PD-1, NKG2A), possibly due to high levels of IL-6 secreted from macrophages during the inflammatory process.

This combination results in a decreased secretion of IFN-γ and decreased degranulation (82, 83). As a result, NK cells may be less able to effectively fight infection when the KIR2DS2*/*HLA-C C1+ group ligand functional unit is not present.

The correct function of the NK cells requires a proper combination of activating and inhibiting KIR genes and their HLA ligands. Due to the defective combinations of *KIR* and HLA, the function of NK cells can be impaired, similar to what has been observed in COVID-19 patients from different ethnic backgrounds (43).

Our study aims to contribute to the understanding of SARS-CoV-2 infection by investigating the role of *HLA-G* polymorphism in the population.

So far, the research has concentrated on how this immunomodulatory molecule plays a role in virus immunopathogenesis in patients with SARS-CoV-2 infection. It has been shown that viruses can upregulate HLA-G molecules in both surface membrane-bound and peripheral soluble forms in cells infected with viruses (53). However, researchers have mainly focused on HLA-G expression and serum levels in COVID-19 patients, rather than focusing on the genetic basis of these manifestations.

The present study is the first to investigate the genetic basis of the *HLA-G* and how it contributes to symptoms associated with SARS-CoV-2 infection. Our first consideration is that the *HLA-G* genetic structure between COVID-19 patients and the controls does not differ significantly. Nevertheless, a significant reduction in the frequency of extended haplotype HLA-G*01:01:01:01/HLA-G/UTR-1 is observed in patients with severe illness (Group S) compared to controls and group A [P< 0.001, Pc =0.002]. On the contrary, our results show that the extended haplotypes HLA-G*01:03:01:02-HLA-G/UTR-5 and HLA-G*01:04:01/04-HLA-G/*UTR-5* are more frequent in Group S than in Group A [P = 0.001, Pc = 0.018 and P< 0.001, Pc = 0.005 respectively].

A recent study has revealed that HLA-G*01:04 and HLA-G*01:03 products have a greater affinity for the heterodimer NKG2A/CD94 receptor than HLA-G*01:01. Therefore, this results in NKG2A to take on a much more potent immunosuppressive action, as it happens in severe COVID-19 infections (53, 84).

In addition, *HLA-G UTR-1* is particularly rare in patients with severe clinical manifestations (**Table 5**). Among the nine polymorphisms that constitute the *HLA-G UTR-1* haplotypes, *14bp Del* (*rs371194629*), and *HLA-G 3’ UTR +3187G* (*rs9380142*) are the most significant ones. Indeed, about 30% of the Caucasian population has these two polymorphisms, which result in close linkage disequilibrium (60).

The “GenOMICC” study has previously conducted genome-wide association studies (GWAS) in 2,244 patients with a critical illness (85). The *HLA-G* 3’ UTR polymorphism *+3187A* (*rs9380142*) was reported as one of the genetic markers associated with severe COVID-19 disease, whereas the *+3187G* variant was not associated with severe disease (85). The study supports the hypothesis that the *HLA-G/UTR-1* haplotype and its polymorphisms, *HLA-G Del* and *HLA-G +3187G*, might play a protective role against severe forms of the disease.

Furthermore, the protective effect is more pronounced in *HLA-G Del/Del* homozygous subjects, making it the most relevant genetic factor in the multivariate analysis (**Table 7**). This genotype, in fact, was found in only 7% of COVID-19 ICU patients (**Figure 1B**).

As several published studies have shown, the *HLA-G Del* polymorphism can result in increased expression of soluble HLA-G molecules by modifying mRNA stability or allowing post-transcriptional regulation (49, 86, 87).

Interestingly, where there was no infection, the levels of soluble HLA-G among patients who recovered from SARS-CoV-2 and those in the control group were not significantly different, even when grouped by their genotype (*Ins/Ins*, *Ins/Del*, and *Del/Del*). Similar results were described by Ali H Ad’hiah, where *HLA-G* genotypes did not significantly affect levels of the soluble molecule in 209 Iraqi patients (88).

However, SARS-CoV-2 infection, like other viral infections, can cause a marked variation in sHLA-G levels due to inflammation and upregulation of immune inhibitory receptors of which HLA-G is a ligand. In addition, a remarkable decrease in HLA-G+ immune cell numbers and exhaustion of host cellular immune responses are commonly observed in patients with severe COVID-19 illness (53).

It is possible that patients with the *HLA-G Del/Del* genotype may express more HLA-G molecules, leading to an increased number of immune-modulatory HLA-G+ cells in the host. In patients with COVID-19 disease, this could reduce the severity by limiting inflammation and cytokine storm responsible for critical cases of illness (89). Moreover, higher levels of sHLA-G are associated with increased expression of sICAM-1 and sE-selectin expression, which may contribute to improved clinical conditions in COVID-19 patients by reducing neutrophil adhesion to activated endothelium (56).

On the other hand, *HLA-G Ins/Ins* genotype has been associated in literature with lower surface expression of HLA-G. This reduction in surface expression may potentially worsen the immune response to viral infections, leading to increased tissue damage, which could explain why this genotype was more commonly observed in ICU patients.

In conclusion, this study is the first to thoroughly investigate the role of *HLA-G* genotypes in SARS-CoV2. Specifically, our results suggest that some *HLA-G* polymorphisms may positively impact the course of COVID-19 through their immunomodulatory effect.

The outcome of SARS-CoV-2 infection is dependent on a complex interaction between the virus and the host, which includes both virus-related factors like the variety of variants and viral load, as well as various genetic and immunological factors of the host. Host genetic factors, including some that have been shown to affect individual susceptibility to develop severe manifestations of COVID-19, should be taken into account along with well-established general risk factors like older age, male gender, and chronic comorbidities when predicting the severity and mortality associated with COVID-19.

It is essential to develop predictive algorithms based on these clinical, genetic, and immunological factors to identify categories of individuals at higher risk of severe short- and/or long-term clinical manifestations in case of SARS-CoV-2 infection. This need is urgent as novel pandemic waves caused by new COVID-19 variants could occur in the coming years (90).

## Data availability statement

The datasets presented in this study can be found in online repositories. The names of the repository/repositories and accession number(s) can be found below: https://www.ncbi.nlm.nih.gov/bioproject/PRJNA904643.

## Ethics statement

The studies involving human participants were reviewed and approved by Ethics Committee of the Cagliari University Hospital; date of approval: May 27th, 2020; protocol number GT/2020/10894. The patients/participants provided their written informed consent to participate in this study.

## Author contributions

All authors contributed to the article and approved the submitted version.
