# Supplementary figures and images for: A review of the main genetic factors influencing the course of COVID-19 in Sardinia: the role of human leukocyte antigen-G
Source: Front Immunol. 2023 Jun 5;14:1138559. doi: 10.3389/fimmu.2023.1138559 (PMC10277491; doi:10.3389/fimmu.2023.1138559)

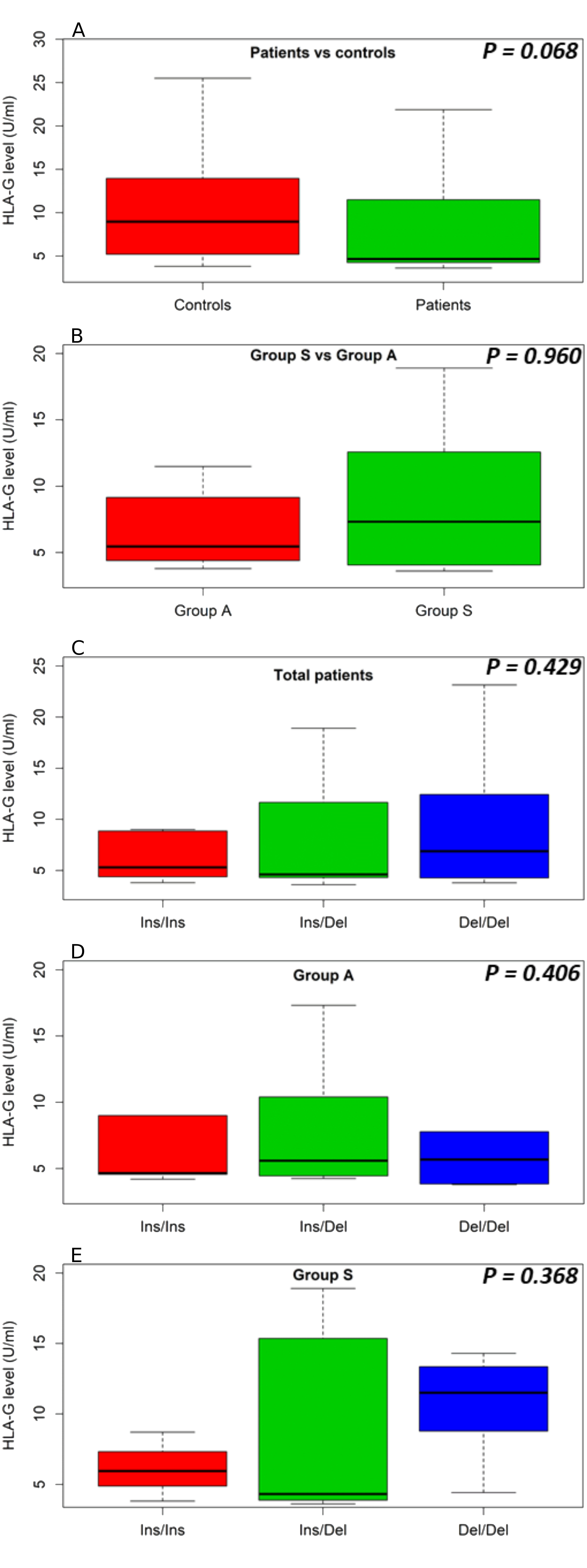

Supplement: Supplementary file 1 [file Image_1.jpeg]
