# Supplementary material for: A review of the main genetic factors influencing the course of COVID-19 in Sardinia: the role of human leukocyte antigen-G
Source: Front Immunol. 2023 Jun 5;14:1138559. doi: 10.3389/fimmu.2023.1138559 (PMC10277491; doi:10.3389/fimmu.2023.1138559)
Supplement: Supplementary file 2 [file Table_1.docx]

**Supplementary Table 1. Allele and Genotype distribution of *rs35044562*, *rs1156361* and *rs11549407* in SARS-CoV-2 patients*.***

| Allele and Genotype distribution | | | | | | |
| --- | --- | --- | --- | --- | --- | --- |
| Gene | SNP | Group A | | | Group S | |
|  |  | (N = 174) | | | (N = 207) | |
| *LZTFL1* |  | Allele | *%* | *X*^2^_HWE_ | *%* | *X*^2^_HWE_ |
|  | *rs35044562* (A>G) | A | 0.933 | 0.0868, | 0.867 | 2.5628, |
|  | (N = 381) | G | 0.066 | *p* = 0.957 | 0.133 | *p* = 0.278 |
|  |  | Genotype | *%* |  | *%* |  |
|  |  | AA | 0.873 |  | 0.739 |  |
|  |  | AG | 0.120 |  | 0.256 |  |
|  |  | GG | 0.006 |  | 0.005 |  |
| *OAS3* |  | Allele | *%* | *X*^2^_HWE_ | *%* | *X*^2^_HWE_ |
|  | *rs1156361* (T>C) | T | 0.379 | 0.9547, | 0.348 | 2.3880 |
|  | (N = 381) | C | 0.620 | *p* = 0.620 | 0.652 | *p* = 0.303 |
|  |  | Genotype | *%* |  | *%* |  |
|  |  | TT | 0.126 |  | 0.097 |  |
|  |  | TC | 0.505 |  | 0.502 |  |
|  |  | CC | 0.367 |  | 0.477 |  |
| *HBB* |  | Allele | *%* | *X*^2^_HWE_ | *%* | *X*^2^_HWE_ |
|  | *rs11549407* (C>T) | C | 0.948 | 0.5177 | 0.991 | 0.0197 |
|  | (N = 381) | T | 0.051 | *p* = 0.772 | 0.009 | *p* = 0.990 |
|  |  | Genotype | *%* |  | *%* |  |
|  |  | CC | 0.897 |  | 0.980 |  |
|  |  | CT | 0.103 |  | 0.019 |  |
|  |  | TT | 0 |  | 0 |  |

*X*^2^_HWE_ =Hardy-Weinberg equilibrium Chi square value; *p* = Hardy-Weinberg equilibrium p value

We calculated the allele and genotype distribution for each single nucleotide variant in both Group A (asymptomatic) and S (moderate and severe patients):

- *rs35044562* (A>G) [chr3:45867532 (GRCh38)] at *LZTFL1* gene, considered the high-risk variant for severe COVID-19 and part of the extended haplotype inherited from Neanderthal (chr3: 45,859,651–45,909,024 (hg19)) .
- *rs1156361* (T>C) [chr12:112938178 (GRCh38)] at *OAS3* gene, associated with protection against severe COVID-19 form and part of the extended Neanderthal haplotype (chr12: 113,350,796 to 113,425,679 (hg19)).
- *rs11549407* (C>T) [chr11:5226774 (GRCh38)] at *HBB* gene, the most common genetic variant responsible for β-Thalassemia in Sardinia.

Genotype and allele frequencies are consistent with Hardy-Weinberg equilibrium for all the considered SNPs in both groups (*p* > 0.05) apart from *rs11549407* (C>T) in Group S.

**Supplementary Table 2. HLA alleles and Haplotypes frequencies compared between Group A and S.**

|  | **COVID-19**  **381 pts** | | **Gruppo A**  **174 pts** | | | **Gruppo S**  **207 pts** | | | **Comparison**  **Group S vs Group A** | |
| --- | --- | --- | --- | --- | --- | --- | --- | --- | --- | --- |
| **HLA alleles and Haplotypes** | **N** | **%** | | **N** | **%** | **N** | | **%** | **P value*** | **OR (95% CI)** |
|  |  |  | |  |  |  | |  |  |  |
| **Partial HLA haplotypes** |  |  | |  |  |  | |  |  |  |
| HLA-A*02:05, B*58:01, C*07:01 | 12 | 0.034 | | 7 | 0.040 | 5 | 0.024 | | 0.394 | 0.59 (0.18 - 1.90) |
| HLA-A*02:05, B*58:01, DRB1*03:01 | 0 | 0.000 | | 0 | 0.000 | 0 | 0.000 | | - |  |
| HLA-A*02:05, C*07:01, DRB1*03:01 | 7 | 0.018 | | 5 | 0.029 | 2 | 0.010 | | 0.254 | 0.33 (0.09 - 1.29) |
| HLA-B*58:01, C*07:01, DRB1*03:01 | 8 | 0.021 | | 7 | 0.040 | 1 | 0.005 | | **0.026** | 0.12 (0.01 - 0.95) |
| HLA-A*02:05, B*58:01 | 13 | 0.034 | | 8 | 0.046 | 5 | 0.024 | | 0.269 | 0.51 (0.17 - 1.60) |
| HLA-A*02:05, C*07:01 | 38 | 0.100 | | 18 | 0.103 | 20 | 0.097 | | 0.865 | 0.93 (0.47 - 1.81) |
| HLA-B*58:01, C*07:01 | 23 | 0.060 | | 12 | 0.069 | 11 | 0.053 | | 0.526 | 0.76 (0.33 - 1.76) |
| HLA-A*02:05, DRB1*03:01 | 23 | 0.060 | | 8 | 0.046 | 15 | 0.072 | | 0.388 | 1.62 (0.67 - 3.92) |
| HLA-B*58:01, DRB1*03:01 | 14 | 0.021 | | 10 | 0.057 | 4 | 0.019 | | 0.058 | 0.32 (0.10 - 1.05) |
| HLA-C*07:01, DRB1*03:01 | 25 | 0.066 | | 11 | 0.063 | 14 | 0.068 | | 1.000 | 1.08 (0.48 - 2.43) |
| **HLA alleles** |  |  | |  |  |  |  | |  |  |
| HLA-A*02:05 | 103 | 0.270 | | 46 | 0.264 | 57 | 0.275 | | 0.818 | 1.06 (0.67 - 1.67) |
| HLA-B*58:01 | 23 | 0.060 | | 12 | 0.069 | 11 | 0.053 | | 0.526 | 0.76 (0.33 - 1.76) |
| HLA-C*07:01 | 92 | 0.241 | | 41 | 0.236 | 51 | 0.246 | | 0.904 | 1.06 (0.66 - 1.70) |
| HLA-DRB1*03:01 | 86 | 0.226 | | 40 | 0.230 | 46 | 0.222 | | 0.902 | 0.96 (0.59 - 1.55) |

*P values were calculated for comparisons between patients with severe clinical manifestations (Group S) and a-paucisymptomatic patients (Group A). Abbreviations: OR = odds ratio; CI = confidence interval;

None of the patients with SARS-CoV-2 infection carried the four loci extended haplotype HLA-A*02:05, B*58:01, C*07:01, DRB1*03:01 which was present in 3.1% of the 420 people selected for the Sardinian population group control [OR = 0.1 (95% CI 0.0 – 0.6), P = 0.002].

**Supplementary Table 3. *KIR* genes and genotype frequencies compared between Group A and S.**

|  | **COVID-19**  **381 pts** | | **Gruppo A**  **174 pts** | | **Gruppo S**  **207 pts** | | **Comparison**  **Group S vs Group A** | |
| --- | --- | --- | --- | --- | --- | --- | --- | --- |
|  | **N** | **%** | **N** | **%** | **N** | **%** | **P value*** | **OR**^#^ **(95% CI)** |
| ***Inhibitory KIR genes*** |  |  |  |  |  |  |  |  |
| **2DL1** | 379 | 0.995 | 172 | 0.987 | 207 | 1.000 | 0.208 | 0.0 (0.0 – 4.5) |
| **2DL2** | 238 | 0.625 | 117 | 0.671 | 121 | 0.585 | 0.089 | 0.7 (0.4 – 1.1) |
| **2DL3** | 354 | 0.929 | 160 | 0.921 | 194 | 0.937 | 0.551 | 1.3 (0.6 – 3.1) |
| **2DL4** | 381 | 1.000 | 174 | 1.000 | 207 | 1.000 | 1 | - |
| **2DL5** | 220 | 0.577 | 105 | 0.605 | 115 | 0.556 | 0.351 | 0.8 (0.5 – 1.3) |
| **3DL1** | 354 | 0.929 | 160 | 0.921 | 194 | 0.937 | 0.551 | 1.3 (0.6 – 3.1) |
| **3DL2** | 381 | 1.000 | 174 | 1.000 | 207 | 1.000 | 1 | - |
| **3DL3** | 381 | 1.000 | 174 | 1.000 | 207 | 1.000 | 1 | - |
| ***Activating KIR genes*** |  |  |  |  |  |  |  |  |
| **2DS1** | 220 | 0.577 | 105 | 0.605 | 115 | 0.556 | 0.351 | 0.8 (0.5 – 1.3) |
| **2DS2** | 222 | 0.583 | 110 | 0.632 | 112 | 0.541 | 0.077 | 0.7 (0.4 – 1.1) |
| **2DS3** | 136 | 0.357 | 58 | 0.336 | 78 | 0.377 | 0.392 | 1.2 (0.8 – 1.9) |
| **2DS4** | 369 | 0.969 | 167 | 0.961 | 202 | 0.976 | 0.394 | 1.7 (0.5 – 6.9) |
| ***- 2DS4 DV+*** | 304 | 0.798 | 140 | 0.803 | 164 | 0.792 | 0.799 | 0.9 (0.5 – 1.6) |
| **- *2DS4 FL+*** | 128 | 0.336 | 48 | 0.276 | 80 | 0.386 | 0.029 | 1.7 (1.0 – 2.6) |
| **2DS5** | 140 | 0.367 | 66 | 0.382 | 74 | 0.357 | 0.671 | 0.9 (0.6 – 1.4) |
| **3DS1** | 154 | 0.403 | 72 | 0.414 | 82 | 0.396 | 0.754 | 0.9 (0.6 – 1.4) |
| ***Genotype*** |  |  |  |  |  |  |  |  |
| **A/A** | 127 | 0.333 | 55 | 0.316 | 72 | 0.348 | 0.586 | 1.2 (0.7 – 1.8) |
| **- *2DS4 DV/DV*** | 76 | 0.199 | 35 | 0.201 | 41 | 0.198 | 1 | 1.0 (0.6 – 1.7) |
| **- *2DS4 DV/FL and FL/FL*** | 51 | 0.134 | 20 | 0.115 | 31 | 0.150 | 0.366 | 1.4 (0.7 – 2.6) |
| **B/x** | 254 | 0.667 | 119 | 0.684 | 135 | 0.652 | 0.586 | 0.9 (0.6 – 1.4) |

*P values and odds ratios were calculated, by means of the Fisher’s exact test, for comparisons between patients with severe clinical manifestations (Group S) and paucisymptomatic patients (Group A). ^#^ The odds ratios express the comparison Group S vs Group A.

Abbreviations: OR = odds ratio; CI = confidence interval; B/x = *KIR* haplotypes AB and BB; *2DS4 DV+ =* deletion variant alleles of the *KIR2DS4* gene; *2DS4 DV/DV* ***=*** homozygous for deletion variant alleles of the *KIR2DS4* gene; *2DS4 DV/FL* and *FL/FL* = heterozygous and homozygous for full-length allelic variants of the *KIR2DS4* gene.

The Table shows the differences between 381 Sardinian COVID-19 patients (divided according to the symptomatology:174 Group A and 207 Group S) for frequencies of activating and inhibitory KIR genes, KIR haplotypes and KIR gene motifs.

**Supplementary Table 4. Comparisons of *KIR* genes and their cognate HLA ligands between COVID-19 patients between Group A and S.**

|  | **COVID-19**  **381 pts** | | **Gruppo A**  **174 pts** | | **Gruppo S**  **207 pts** | | **Comparison**  **Group S vs Group A** | |
| --- | --- | --- | --- | --- | --- | --- | --- | --- |
|  | **N** | **%** | **N** | **%** | **N** | **%** | **P value** | **OR (95% CI)** |
| ***KIR Ligands*** |  |  |  |  |  |  |  |  |
| **C1/C1** | 89 | 0.234 | 39 | 0.224 | 50 | 0.242 | 0.717 | 1.1 (0.7 – 1.8) |
| **C2/C2** | 110 | 0.289 | 48 | 0.276 | 62 | 0.300 | 0.651 | 1.1 (0.7 – 1.8) |
| **C1/C2** | 181 | 0.475 | 87 | 0.500 | 94 | 0.454 | 0.410 | 0.8 (0.5 – 1.3) |
| **HLA-Bw4** | 285 | 0.748 | 131 | 0.750 | 155 | 0.749 | 1 | 1.0 (0.6 – 1.6) |
| **- Bw4 Ile80** | 246 | 0.646 | 119 | 0.684 | 127 | 0.614 | 0.163 | 0.7 (0.5 – 1.1) |
| **-Bw4 Thr80** | 92 | 0.241 | 41 | 0.237 | 51 | 0.246 | 0.904 | 1.1 (0.6 – 1.8) |
| **HLA-Bw6** | 322 | 0.845 | 142 | 0.816 | 180 | 0.870 | 0.158 | 1.5 (0.8 – 2.7) |
| ***Activating KIRs/HLA ligands*** |  |  |  |  |  |  |  |  |
| ***2DS1*/HLA-C2 group** | 176 | 0.462 | 84 | 0.480 | 92 | 0.444 | 0.472 | 0.9 (0.6 – 1.3) |
| ***2DS2*/HLA-C1 group** | 110 | 0.289 | 63 | 0.362 | 47 | 0.227 | **0.005** | **0.5 (0.3 – 0.8)** |
| ***2DS4*/HLA-A*11 or -C*04** | 142 | 0.373 | 64 | 0.368 | 78 | 0.377 | 0.915 | 1.0 (0.7 – 1.6) |
| ***3DS1*/HLA-Bw4 epitope** | 112 | 0.294 | 53 | 0.303 | 59 | 0.285 | 0.735 | 0.9 (0.6 – 1.5) |
| ***Inhibitory KIRs/HLA ligands*** |  |  |  |  |  |  |  |  |
| ***2DL1* /HLA-C2 group** | 288 | 0.756 | 133 | 0.763 | 155 | 0.749 | 0.811 | 0.9 (0.6 – 1.5) |
| ***2DL2* /HLA-C1 group** | 118 | 0.310 | 70 | 0.402 | 48 | 0.232 | **3.7 ⋅ 10^-4^** | **0.4 (0.3 – 0.7)** |
| ***2DL3* /HLA-C1 group** | 236 | 0.619 | 105 | 0.605 | 131 | 0.633 | 0.597 | 1.1 (0.7 – 1.8) |
| ***3DL1* /HLA-Bw4 epitope** | 266 | 0.698 | 121 | 0.697 | 145 | 0.700 | 1 | 1.0 (0.6 – 1.6) |

Different combinations of activating and inhibitory *KIR* genes with their ligands were analyzed and compared, by means of the Fisher’s exact test, between patients with a-paucisymptomatic patients (Group A) and patients with severe disease (Group S).

Abbreviations: OR = odds ratio; CI = confidence interval. The Table shows the differences between 381 Sardinian COVID-19 patients (divided according to the symptomatology:174 Group A and 207 Group S) for the combination of *KIR* locus with their respective *HLA* ligands (KIR-HLA functional units).

**Supplementary Table 5. P values for all the possible comparisons between the *HLA-G* polymorphism and genotypes in the groups of controls and patients.**

|  | **P value** | | | | | |
| --- | --- | --- | --- | --- | --- | --- |
|  | **A vs Ctr** | **S vs Ctr** | **S vs A** | **ICU vs Ctr** | **ICU vs A** | **ICU vs S*** |
| ***Del* or *Ins*** | 0.339 | 0.119 | 0.035 | 0.001 | 0.0004 | 0.006 |
| ***Ins/Ins* or (*Ins/Del* and *Del/Del)*** | 0.326 | 0.920 | 0.379 | 0.032 | 0.011 | 0.006 |
| ***Ins/Del* or (*Ins/Ins* and *Del/Del)*** | 0.787 | 0.041 | 0.147 | 0.672 | 0.761 | 0.427 |
| ***Del/Del* or (*Ins/Ins* and *Ins/Del)*** | 0.537 | 0.010 | 0.008 | 0.001 | 0.0009 | 0.034 |

Ctr = Control group, A = Asymptomatic or paucisymptomatic patients, S = Patients with severe symptoms, ICU = Patients admitted in Intensive Care Unit, S* = patients with severe symptoms but not admitted in ICU. The table reports the P values for the comparisons between the frequencies of the *HLA-G* 3’UTR 14-bp polymorphism (*Ins* or *Del*) and the *HLA-G* genotypes (*Ins/Ins*, *Ins/Del* and *Del/Del*) in the control sample (Ctr) and in several groups of patients (A, S, ICU and S*).

**Supplementary Figure 1.** Soluble HLA-G Plasma Level was measured in controls and patients six months after healing. P values were calculated using the Wilcoxon rank sum test for comparisons between two groups (Fig. 1A and 1B) and the Kruskal-Wallis rank sum test for comparisons between three groups (Fig. 1C, 1D and 1E). A) Comparison between sHLA-G levels in controls and COVID-19 patients. B) Comparison between sHLA-G levels in Group A (paucisymptomatic) and Group S (Severe). Panel C, D, E: difference of sHLA-G levels in patients divided according to the genotype group: homozygous (Ins/Ins), heterozygous (Ins/Del) and homozygous (Del/Del).
